# Digital health intervention reconnects war-affected people living with HIV to healthcare: Ukraine case study

**DOI:** 10.1093/oodh/oqaf001

**Published:** 2025-01-08

**Authors:** Hlib Aleksandrenko, Maryna Shevchenko, Olga Chervak

**Affiliations:** School of Public Health, National University of Kyiv–Mohyla Academy, 2 Skovorody St., Kyiv, 04655, Ukraine; School of Public Health, National University of Kyiv–Mohyla Academy, 2 Skovorody St., Kyiv, 04655, Ukraine; Communication Department, Pact Ukraine, 49a Volodymyrska St., Kyiv, 01001, Ukraine

**Keywords:** digital health, digitalization, healthcare management, public health, universal health coverage, health system resilience

## Abstract

The unprovoked full-scale invasion of Ukraine in February 2022 severely damaged the national health system, disconnecting thousands of people living with human immunodeficiency virus (PLHIV) from essential health services. Digital health interventions (DHIs) offer the potential to reconnect war-affected PLHIV to healthcare and improve health system resilience in case of emergencies. This study aimed to present a comprehensive case study of the implementation and lessons learned from a DHI aimed at reconnecting Ukrainian PLHIV to healthcare in the context of war. A DHI called ‘#ARTporuch’ was implemented in Ukraine in response to the war-related challenges for PLHIV. The case study methodology was used to present the DHI’s life cycle, from creation to maintenance. Usage and geographic coverage data were analyzed descriptively. The multi-component DHI, consisting of a website, chatbot, database and information campaign, was implemented. The case study yielded pivotal insights into the DHI lifecycle, including rapid conceptualization, security considerations, agile implementation and continuous adaptation. The online advertising generated >10 million impressions within the information campaign. The website attracted 20 619 visitors, while the chatbot was activated by 2950 users. This case study underscores the potential of DHI as a crisis-response component to reconnect PLHIV to healthcare during wartime. While quantitative evidence of effectiveness is limited due to wartime constraints, the study provides insights into the implementation of DHI in conflict-affected or resource-constrained settings, contributing to the ongoing efforts to achieve universal health coverage and enhance health system resilience planning.

## Lay Summary

The ongoing war in Ukraine has severely disrupted the country’s health system, making it difficult for thousands of people living with human immunodeficiency virus to access life-saving treatments. To address this urgent issue, we developed a digital health intervention called #ARTporuch, which translates to ‘Antiretroviral Therapy Nearby’ in Ukrainian. This intervention includes a chatbot, a website and an information campaign aimed at reconnecting war-affected people with the nearest functioning health facilities where they can continue their human immunodeficiency virus treatment.

Our study examined how #ARTporuch was developed, implemented and used during wartime. The intervention reached millions through online advertising. Over 20 000 people from Ukraine and 57 other countries used the website. Nearly 3000 people used the chatbot to find health facilities and access essential services.

#ARTporuch demonstrated how simple, yet timely and accessible digital solutions can help maintain access to healthcare during the war. It provided up-to-date information on available services, both domestically and abroad. The intervention’s adaptability was crucial as it expanded to meet the emerging needs of people living with human immunodeficiency virus who fled the country.

Our findings show that we can use digital health interventions to ensure that no one is left behind in their right to health, even in emergencies such as war. The lessons learned from the #ARTporuch intervention can inform the creation and adoption of similar solutions to build health system resilience and achieve universal health coverage.

## INTRODUCTION

### Background

The full-scale invasion of Ukraine in February 2022 forced millions of Ukrainians to leave their homes and become internally or externally displaced people. The national health system has been severely damaged, with the infrastructure of facilities destroyed, human resources lost and logistical routes for the supply of essential medicines disrupted [1]. In just 15 months since the beginning of the war, international experts have recorded >1000 attacks on Ukraine’s health system [2]. As a result, people’s health state and access to health services, especially those living in areas of active hostilities, have significantly deteriorated. Furthermore, it has led to the disconnection of thousands of people living with human immunodeficiency virus (PLHIV) from the health services they rely on for survival [3]. As of the beginning of 2022, >129 000 PLHIV were receiving antiretroviral therapy (ART) and were at risk of treatment interruption [4]. On a monthly basis, >3000 PLHIV who are internally displaced persons (IDPs) are compelled to seek health services in a new place of residence as a consequence of the ongoing migration [5]. Reconnecting people to health care became a priority for health system recovery in Ukraine [6]. This situation has highlighted the need for innovative approaches and tools to ensure the recovery and accessibility of health services for the population, as well as to increase the responsiveness and resilience of the health system in wartime. Digital health can open new opportunities to address these issues [7–10].

Digital health interventions (DHIs) can potentially improve the accessibility and quality of health services [11]. According to WHO experts, DHIs refer to any digital product, service or tool for delivering health services and information to individuals, groups and entire health systems [12]. Moreover, DHIs are increasingly recognized to have significant potential to improve health system strengthening and health outcomes [13]. A body of evidence shows the benefits of digital health solutions in various contexts worldwide [14, 15]. Studies emphasize the ability of DHIs to expand service reach, enable remote care delivery, streamline processes, and strengthen health data collection and analysis [16, 17]. However, the literature indicates significant variability in the success of DHIs, depending on contextual factors and implementation challenges [18–20]. This statement highlights the necessity for additional research and evaluation of DHIs in various settings.

There is evidence indicating that DHIs can enhance the resilience and responsiveness of health systems during crises, such as the COVID-19 pandemic. Telehealth, for instance, played a critical role in delivering care, particularly to vulnerable populations [21]. Commonwealth countries have effectively utilized digital tools to strengthen health systems, engage communities, manage emergencies and leverage data [22, 23]. Moreover, DHIs are pivotal in improving adherence to antiretroviral therapy among PLHIV, thus addressing a significant global health challenge [24, 25]. Nevertheless, further studies are necessary to assess the efficacy of DHIs in reconnecting PLHIV to healthcare [26]. Additionally, there is a gap in research regarding the utilization of DHIs in emergencies related to conflicts and analyzing the associated challenges.

Ukraine’s ongoing painful experience offers a significant opportunity to build the evidence base regarding DHI’s emergency response potential. Recent studies show the resilience and adaptability of the Ukrainian healthcare system in the face of adversity, mainly through digital solutions [27]. Studies on DHIs in resource-constrained settings during emergencies offer insights and lessons that can guide the use of adaptable and far-reaching DHIs to enhance global crisis resilience.

Therefore, there is an urgent need to monitor and evaluate the effectiveness of DHIs as a response to reconnecting people to healthcare in the context of war.

### Objectives

The purpose of this article is to present a comprehensive case study of the implementation and lessons learned from a DHI aimed at reconnecting Ukrainian PLHIV to healthcare in the context of war.

The results of the study are expected to contribute to better decision-making in DHI deployment strategies.

## METHODS

### DHI description

The article presents an analysis of the development and implementation of the DHI, named ‘#ARTporuch,’ created to address the unique challenges of the ongoing war in Ukraine. It aims to reconnect war-affected PLHIV to antiretroviral therapy, as well as other medical and social services both in Ukraine and abroad [28, 29]. The DHI was launched as part of a national information campaign to improve access to HIV services and ART adherence. The campaign was implemented by the State Enterprise Center of Public Health of the Ministry of Health of Ukraine (the CPH of the MoH of Ukraine) with the support of the USAID Community Action for HIV Control Project (the USAID CAHC project).

The WHO Classification of Digital Interventions, Services and Applications in Health [30] was employed to classify the DHI. The intervention addressed Health System Challenges related to Information (1.3, 1.5), Availability (2.2) and Utilization (5.2, 5.4), particularly for PLHIV during the war. The targeted primary users for the DHI are people for whom it enables access to health information (1.6.1). These were delivered through Service and Application types such as Communication systems (A1), Telehealth systems (A9) and Analytics Systems (D1).

This DHI was developed between March and April 2022 and launched in mid-April 2022.

### Case study methodology

A case study approach was adopted to provide a thorough understanding of the ‘#ARTporuch’ DHI implementation process. This methodology was chosen to offer an in-depth, contextual analysis of the DHI’s development and implementation, capturing valuable insights into the challenges and solutions encountered in an emergency war setting.

Data for the case study were collected from internal protocols, project documentation and experiential knowledge gained throughout the DHI implementation process. This approach allowed for a rich, detailed account of the intervention’s lifecycle, capturing the technical aspects and the contextual factors influencing its development and deployment.

**Figure 1:**
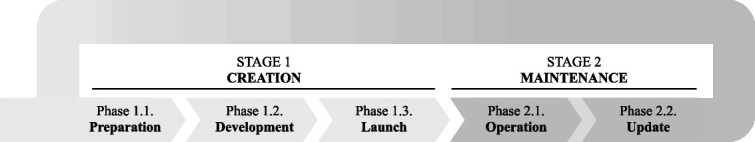
DHI lifecycle framework

The case study description is structured according to the key stages and phases of the DHI implementation, following an author-proposed DHI lifecycle framework (see Fig. 1). This framework provides a systematic approach to analyzing the intervention’s creation and ongoing maintenance, allowing for a comprehensive understanding of the DHI’s evolution over time.

Moreover, it presents the results of the DHI implementation, including information campaign, usage statistics and geographical coverage data. These results offer a comprehensive view of the DHI’s reach and utilization.

The WHO mHealth Evidence Reporting and Assessment guidelines [31] were applied to ensure comprehensive and transparent reporting of the DHI.

### Data collection and analysis

The results of the DHI implementation from April 2022 to June 2023 were analyzed using relevant indicators based on actual data from chatbot and website usage. Data for the website were available for each month during the implementation period. The Google Ads information campaign ran from April to June 2022, with the highest activity in May 2022, and only the results as of the end of the period were analyzed. For the chatbot, data analysis was conducted for the selected periods of April, July, December 2022 and June 2023. This was necessary due to the cumulative collection of data in the chatbot and the need for valid data for analysis.

Descriptive statistics were employed for data analysis, which aligned with the study’s purpose. The data source comprised databases of digital products and the results of the information campaign from Google Ads. Individual depersonalized datasets were obtained in a coded format from the Project. Data analysis and interpretations were carried out using Google Analytics, Looker Studio and Google Spreadsheets.

### Ethical, privacy and security considerations

The study was conducted by the current legislation of Ukraine on personal data protection and was approved by the Research Ethics Committee of the National University of Kyiv–Mohyla Academy on 14 November 2023 (protocol number 5). The use of depersonalized data and implementation experience from the USAID CAHC project was officially approved (see Supplementary Material 1). Using the chatbot and website required users to agree to the terms of use and privacy policy, allowing for the use of their anonymized data for analysis and service improvement. All user information was securely stored and anonymized to prevent identification. The research adhered to the ethical principles of the Belmont Report and the institutional policies of the National University of Kyiv–Mohyla Academy.

## RESULTS

### Creation stage

#### Preparatory phase

The urgent need for intervention arose in March 2022, after the initial shock of the full-scale invasion had subsided. It became clear that a response was needed to address the multiple challenges faced by the Ukrainian population in the context of martial law. The USAID CAHC project took the initiative to support the CPH of the MoH of Ukraine and played a driving role in implementing a DHI to support PLHIV in these highly challenging conditions.

The initial and most crucial phase of the DHI implementation process entailed preparation and conducting a situational analysis to evaluate the existing circumstances for PLHIV. The analysis revealed a series of significant challenges that impeded the target population’s ability to access essential health services. As indicated by the CPH of the MoH of Ukraine, the HIV service system has encountered significant challenges since the onset of the full-scale invasion, including the migration of the population [5]. The patient’s route has undergone a significant alteration, and the connection with the physician and the health facility (HF) where the patient is under medical supervision has been severed. The disruption of transportation has resulted in the untimely delivery of the primary stock of medicines and medical supplies. This has precipitated a critical situation regarding the provision of treatment and the potential for the interruption of HIV treatment throughout the country, as well as the monitoring of treatment effectiveness. These circumstances have culminated in a critical situation in which the continuous receipt of vital medicines and other health services has become increasingly challenging for PLHIV.

Notwithstanding the active involvement of volunteers and international and public organizations operating under martial law, the principal challenge of information uncertainty and the absence of up-to-date information support for PLHIV remains unresolved. Ultimately, reliable and timely information regarding the functionality of HFs offering essential health services was of paramount importance in facilitating the reconnection of PLHIV to healthcare. Accordingly, the subsequent logical step was to analyze the existing solutions designed to address the problem of information asymmetry in this area. Among the services provided was #HELPnow, which facilitated the receipt of antiretroviral therapy, anti-tuberculosis drugs, drugs for the treatment of viral hepatitis and substitution maintenance therapy [5]. This solution partially assisted in overcoming the existing obstacles to the continuous treatment of socially problematic diseases for vulnerable populations. The service’s architecture permitted users to submit requests via various digital communication channels, and each request was processed by a responsible coordinator, who prepared and sent the necessary response. Due to its complete dependence on the manual work of a specialist to process each individual request, its analogous nature required significant time resources and made it difficult to scale. Therefore, there was an urgent need for a more innovative architecture with automated request processing to increase response time and the ability to serve a growing number of users without requiring additional, minimal human resources.

Following the collection of baseline information, situation analysis and the completion of a solutions analysis, the process of developing the intervention concept commenced. The process was conducted by the USAID CAHC project’s working group, comprising a technical expert, a communications specialist and other relevant specialists as required. The primary stakeholder was the CPH of the MoH of Ukraine, an institution entrusted with the coordination and implementation of state policy in the domain of addressing the HIV and acquired immunodeficiency syndrome (AIDS) epidemic. Subsequently, further work on the concept was conducted in close collaboration with this and other key stakeholders and interested parties. The proposed concept was based on the provision of up-to-date information to the target audience regarding the availability and accessibility of health services for the target population, including antiretroviral therapy, in a convenient and automated manner.

The CPH of the MoH of Ukraine had already closed up-to-date information on active HFs providing essential health services to PLHIV. The main objective was to disseminate this information to the target audience. Consequently, at the inaugural strategic meetings with key stakeholders, the potential for developing a digital solution—a web platform for the dissemination of this information—was investigated. The primary objective of this web platform was to serve as an interactive map displaying the precise locations of HFs offering essential services. The utilization of existing solutions, such as Google Maps, was not a viable option, as the CPH of the MoH of Ukraine had prohibited the mapping of critical infrastructure due to the potential risk of shelling. Considering the significant shelling of critical infrastructure, the publication of precise geolocation data regarding existing HFs in the public domain posed a considerable risk to the safety of these facilities. In response to these challenges, the initial concept was adapted to align with the evolving operational landscape. To this end, a series of strategic meetings were conducted with partners, and the team engaged in internal discussions to identify alternative solutions. The key principles underlying the proposed intervention concept were safety, responsiveness, flexibility and ease of use.

**Figure 2:**
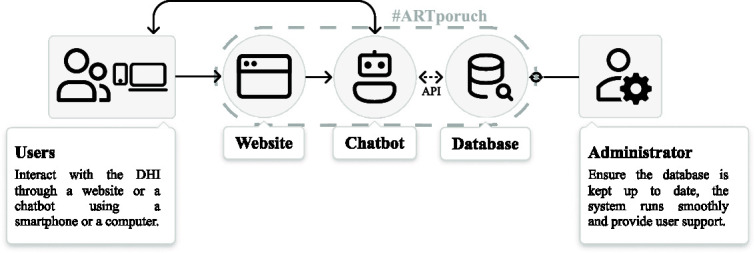
General view of the DHI ‘#ARTporuch’ architecture

Consequently, a novel and enhanced digital solution concept was devised, integrating multiple products: a website, a chatbot in Telegram Messenger, and a discrete database that comprises data on active HFs offering the requisite services (see Fig. 2). This approach allowed combining the advantages of different channels of communication with the audience into a single, automated and flexible architecture. The aforementioned components were integrated into a unified intervention, designated as ‘#ARTporuch’ (‘poruch’ denoting nearby).

The objective of the chatbot was to allow users to find the nearest HFs and points to receive HIV treatment and other necessary support based on their individual needs and location. The chatbot’s functionality was planned to include three search options: location-based search in Ukraine (searching by the user’s longitude and latitude for the nearest HF), oblast-based search (searching for all HFs in the selected territorial unit—oblast) and abroad (searching for points that provide the necessary health services in the selected country abroad). To obtain up-to-date information on HFs, the chatbot was intended to be connected via webhook or ‘event-driven APIs’ to a separate database maintained and updated in real time by the CPH of the MoH of Ukraine, who served as the administrator. For security purposes, it was decided that users would be required to authorize the chatbot using a national phone number and provide geolocation information to find the nearest HFs in Ukraine. This measure was deemed necessary due to the extensive shelling of critical infrastructure in Ukraine and to ensure that users were not from the aggressor country. Additionally, the chatbot was designed to provide links to access information about HIV treatment, automatically answer common questions about antiretroviral therapy and offer support options through an online chat with the coordinator of a national HIV/AIDS hotline or administrator, all without requiring authorization. The decision to employ a chatbot as a pivotal element was influenced by the favorable outcomes observed by the global organization FHI360 in utilizing analogous solutions in the domain of HIV prevention [32]. Secondly, the chatbot provided partial access to the information already received, even without an internet connection.

The website’s objective was to function as an online platform, aggregating stakeholders’ detailed open information on medical and social support for PLHIV and information on humanitarian aid in Ukraine and abroad. Furthermore, it was anticipated that the website would include a link to the chatbot, allowing users to transition from the website to the chatbot seamlessly.

It was of great importance that the concept was not limited to a textual or verbal description; rather, it was developed into a website mockup and a chatbot prototype with presentation functionality. The availability of such prototypes enabled the visualization of the anticipated outcomes of the product while discussing the concept in the working group and with stakeholders. This facilitated a more comprehensive understanding of the proposed ideas and the capacity to expeditiously implement necessary alterations and enhancements during the initial stages of development. Furthermore, the completion of the prototypes imbued the product concept itself with greater substance and persuasiveness for the stakeholders, thereby fostering their willingness to collaborate more assuredly in this project, which was of paramount importance.

#### Development phase

In mid-May 2022, after the concept of the multi-component DHI was agreed and approved, the actual stage of its creation began. The development involved several successive stages. At the first stage, based on the previously prepared concept and existing developments, a basic chatbot algorithm, website terms of reference and general database architecture were formed. Further work on the development of individual digital components (chatbot, website, database) was based on agile product management [33, 34]. This approach enabled us to respond expeditiously to new requirements and changes that inevitably arose during product development. For instance, a subsequent task is to create a database and prepare data on HFs. This scope of work was not adequately estimated during the preparation phase, necessitating a rapid adaptation. It was essential to enhance the description of HFs with a user-oriented approach and incorporate the longitude and latitude data required for the chatbot search function.

A feature of the first stage of development was the formation of a detailed user scenario that was developed based on the principles of human-centered design, with the active participation of key stakeholders as subject-matter experts. In particular, one of the key functionalities was the integration between the website and the chatbot to provide an end-to-end user experience. The user could start interacting on the website and then go directly to the chatbot at a convenient time without losing the context of the previous steps. For example, if a user indicated his or her status as an internally displaced person on the website when he or she went to the chatbot, he or she would immediately get access to the search function, subject to a short registration. This approach allowed us to optimize the user journey, saving time and effort for the target audience of the products.

The issue of selecting the optimal technology stack for DHI development was addressed separately. After a detailed analysis of market requirements and opportunities, three main options were formed: (i) developing custom code, (ii) using ready-made open-source solutions and (iii) utilizing no-code and low-code platforms. After a comparative analysis of the advantages and disadvantages of each of the three considered technological implementation options, considering the requirements and possibilities of the project, it was decided to use no-code and low-code tools. This approach would allow for the speeding up of development and reducing its cost by using off-the-shelf components and the possibility of rapid prototyping. However, an important criterion in choosing a no-code and low-code platform provider, as well as an important security criterion, was the absence of ties with the aggressor country. The development phase included architecture, technological implementation and user interface and user experience (UI/UX) design.

Additionally, this phase included planning for monitoring usage data and facilitating timely adjustments to the DHI. Table 1 provides a list and description of the indicators developed to analyze the results of the DHI implementation.

**Table 1:** DHI implementation analysis indicators

Group	Name	Description
Promotion (communication)	Contextual Google Ads	Advertising results among a target audience that sees advertising banners while browsing the Internet
	Discovery Google Ads	Advertising results that appear in the YouTube app, the Google app and the Gmail app
	Search Google Ads	Advertising results on web pages that display results from search engine queries
Website indicators	Audience	Total number of website users, sessions, new users, average session duration, etc.
	Geography	Geographic distribution of website users
	Referrals to other online resources/channels	Number and percentage of clicks on links from the website to other online resources, etc.
Chatbot indicators	Activation and authorization	Number of activations, authorizations, percentage of authorized chatbot users
	Actual location	Distribution of website users by geography (in Ukraine or abroad)
	IDPs	Data on the location of chatbot users—at home or away from home (for those in Ukraine)
	Information on the operational status of HFs in Ukraine	Information on the actual availability of information to chatbot users about the work of their HF (for those at home in Ukraine)
	Ability to visit HFs in Ukraine	Information on the user’s ability to physically reach the HF (for those in Ukraine and at home)
	Search function	Use of search options within Ukraine or abroad
	Search in Ukraine	Search distribution by oblast in Ukraine
	Search abroad	Search distribution by country
	Getting the necessary services	Actual visits to HFs and/or getting necessary services based on the chatbot’s information
Cross-cutting indicators	User feedback, monitoring results	Analyzing feedback and results to improve the products
	HIV/AIDS hotline	Usage of HIV/AIDS hotline contacts from the website and/or online chat in the chatbot

An important next step was also assigned to testing both intermediate development results and final products before the pilot launch. The pre-pilot testing involved members of the internal team and externally verified volunteers (who were selected based on convenience from among colleagues) to speed up the process as much as possible due to the limited timeframe.

#### Launch phase

In early April 2022, after creating a minimum viable product (MVP) with basic functionality, it was piloted to collect user feedback and further adapt and refine the solution. At the MVP stage, only the chatbot and the database were operational, while the launch of the website and the information campaign were temporarily postponed until the initial testing stages were completed. This decision was based on the understanding that the website and information campaign played a supporting role in promoting and providing access to the chatbot, which was the main component of the product and directly interacted with the end user, delivering necessary information. In addition, the chatbot had a more complex architecture, which was expected to require more attention during the launch. One challenge that emerged during the pilot phase of the chatbot was the difficulty in engaging with the target population to obtain data on their experiences and feedback. To address this, links to the chatbot were organized and distributed in PLHIV-specific channels and chats provided by volunteers. User interaction with the chatbot was logged in the administrative panel, which made it possible to track the route according to the algorithm and connect to the administrator for support. After this, additional testing was carried out, the chatbot was refined based on the results obtained and then a pilot launch of the website with similar goals was carried out. After the pilot launch of the website, we also analyzed the relevant web analytics metrics, including data on the number and sources of visits to the resource, the depth of browsing, and the buttons and links that most often led to the chatbot. All the information received during the pilot launch of the website was processed daily and used to refine and optimize the product.

After completing the piloting and testing cycle in mid-April 2022, the website was launched in full. At the same time, an information campaign was launched to communicate the product and its value to the representatives of the target population—PLHIV [35]. Both online and offline marketing approaches were utilized to attract users to the website and/or chatbot as part of the information campaign. The focus was primarily on online advertising, specifically Google Ads. The information campaign covered only the government-controlled areas of Ukraine.

### Maintenance stages

#### Operation phase

After the full launch of the integrated product in mid-April 2022, its functionality and performance were continuously analyzed based on key indicators along the user journey. Feedback from direct users, data on their experience of using the DHI, performance indicators of individual components and the DHI as a whole were also logged, summarized and analyzed. Depersonalized user data were regularly downloaded, updated and used for reporting, identifying technical deficiencies or user needs, and planning further updates.

Despite the comprehensive automation of the solution, users encountered difficulties in obtaining the requisite assistance through the available functionality. In particular, there were instances where assistance was required for individuals in non-government-controlled areas. These issues were reviewed on a case-by-case basis and referred to the relevant partner organizations.

Additionally, some users have requested technical assistance for reasons that were not immediately apparent. The analysis of such cases indicated that the user was unable to use the chatbot due to a low level of digital literacy. This led to the need for minor but urgent changes and the inclusion of additional guidance in the chatbot design. To illustrate, the user bypassed the preliminary stages of the onboarding process and entered their address into the chatbot, anticipating a prompt response with the nearest information about the nearest HF. However, this function was not enabled due to a lack of interaction with the designated button within the menu.

#### Update phase

Updating is an integral part of the implementation of a DHI if it plans to be sustainable and relevant, and this requires the availability of usage data and change management. Based on the results of regular analysis, the need was identified to upgrade the functionality of the DHI with additional features related to barriers within up-to-date information on the availability and conditions for obtaining necessary services by Ukrainian PLHIV who were abroad due to full-scale invasions. In response to the identified demand, it was initiated to expand the DHI’s functionality. In particular, a situational analysis and a series of strategic meetings with key stakeholders were conducted. According to WHO forecasts, the number of PLHIV who ended up abroad could be as high as 30 000. Poland, the Czech Republic, Germany, Moldova, Slovakia and Romania have borne the greatest burden. However, there were no accurate statistics on the number of PLHIV who received ART in Ukraine and crossed the border due to closed registers. Based on the results of this preparatory work, a new version of the product was developed and implemented with additional features to search for up-to-date information on the availability of vital treatment for Ukrainian PLHIV who have relocated abroad due to the full-scale invasion. The principle of operation of the updated version remained unchanged; a separate register was created with data on the facilities of health service provision to PLHIV abroad, which was constantly updated.

Further maintenance of the DHI was done without significant updates. In early 2023, due to the partial improvement in the availability of services for PLHIV, it was decided that the DHI should be maintained until mid-2023, with a subsequent review of its need and necessity to determine whether to continue or discontinue it. However, the initiatives of the USAID CAHC project and the CPH of the MoH of Ukraine were also concentrated on preventive measures, including HIV testing.

### DHI results

#### Information campaign

Based on the Google Ads results presented in Table 2, discovery advertising was found to be the most effective. However, excluding the first week of testing from the period, the click-through rate for search advertising is 11%, indicating that every 10 people visited the website or chatbot.

**Table 2:** Information campaign performance key indicators by type of advertising

Type of Google Ads	Impressions	Clicks	Click-through rate (%)
Contextual	10 400 000	62 700	1
Discovery	374 214	17 087	5
Search	177 000	2970	2
Total	10 951 214	82 757	1

#### Website

**Figure 3:**
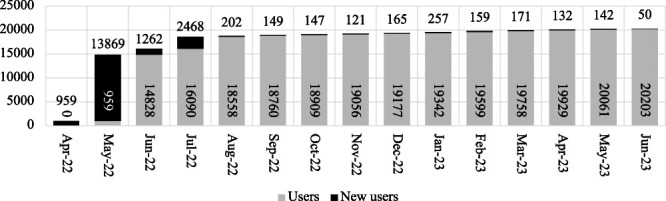
Website audience by periods and types of users

During the implementation period, the website had a total of 20 253 users (Fig. 3). The most significant increase in new users occurred in May 2022, with 13 869 users. Users spent 22 s on the website on average, and the average number of sessions per user was 1.09.

**Figure 4:**
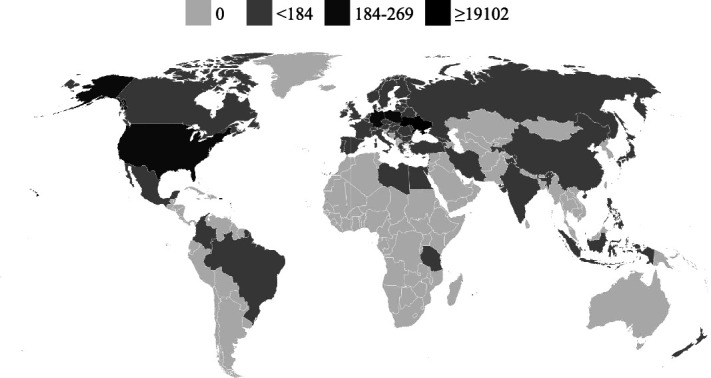
Website user geography divided by the number of visitors

The analysis of the website’s user geography revealed that most users were based in Ukraine, with 19 102 users (almost 92% of the total) accessing the website from Ukraine. However, there was also an increase in the number of users from 57 foreign countries. Most of the visitors came from Poland, the USA and Germany (Fig. 4).

#### Chatbot

Based on the data presented in Table 3, there were 2950 chatbot users at the end of the analysis period. The largest increase in new users was observed between April and June 2022, which correlates with the growth in the number of users on the website in May 2022. The proportion of authorized users remained stable at ~30%. Of the total number of users, 51% stated that they were located in Ukraine, while 38% of users stated that they were located abroad. However, the number of users from abroad increased from 30% at the beginning of the period to 39% at the end of the period. At the same time, the number of IDPs among chatbot users decreased, first increasing from 15% to 21% in April–June 2022, then decreasing to 20% in December 2022 and 19% in June 2023.

**Table 3:** Chatbot performance key indicators

Name	Disaggregation	Apr. 2022	Jul. 2022	Dec. 2022	Jun. 2023
Activation and authorization	# of users who have activated the chatbot	854	2051	2510	2950
	# of users authorized after activation	244	621	754	880
	% of authorized users	29	30	30	30
Actual location	# of users who activated the chatbot abroad	256	711	937	1136
	# of users who have activated the chatbot in Ukraine	483	1103	1300	1493
	# of users who did not specify their location	115	236	275	323
IDPs	# of users who are not at home^a^	125	427	493	551
	# of users who are at home^a^	277	616	716	786
	# of users who did not provide information on internal displacement	81	60	91	156
Information on the operational status of HFs in Ukraine	# of users who do not know the work status of their HF	193	387	455	545
	# of users who know that their HF is working	61	111	128	152
	# of users who know that their HF is not working	15	42	52	55
	# of users who did not indicate the availability of information on the work status of their HF	8	76	81	34
Ability to visit HFs in Ukraine	# of users who cannot reach their HF	11	29	37	39
	# of users who can reach their HF	4	10	12	12
	# of users who did not indicate whether they could reach their HF	0	3	3	4
Search function (Ukraine and abroad)	# of users who used the location search option in Ukraine and received a result	54	171	224	247
	# of users who used the oblast search option and received a result	194	481	579	683
	# of users who used abroad search option and received a result	N/A^b^	219	393	503
Getting the necessary services	# of users who successfully reached an HF based on the search results and/or received the necessary services	48	213	257	294
	# of users who did not reach an HF and/or received the necessary services based on the search results	33	127	158	182
	# of users who have not yet reached an HF and/or received the necessary services based on the search results	17	110	159	224

^a^At home—current place of living before war.

^b^Until 12 May 2022 information was not available, users received the necessary contacts and information for further self-search for HIV treatment abroad.

During the period, the percentage of users in Ukraine who reported to the chatbot that they lacked information about the work status of their HFs due to war-related issues decreased from 23% to 18%. Meanwhile, the percentage of users who were unable to reach their HFs increased from 1% to 4%.

**Figure 5:**
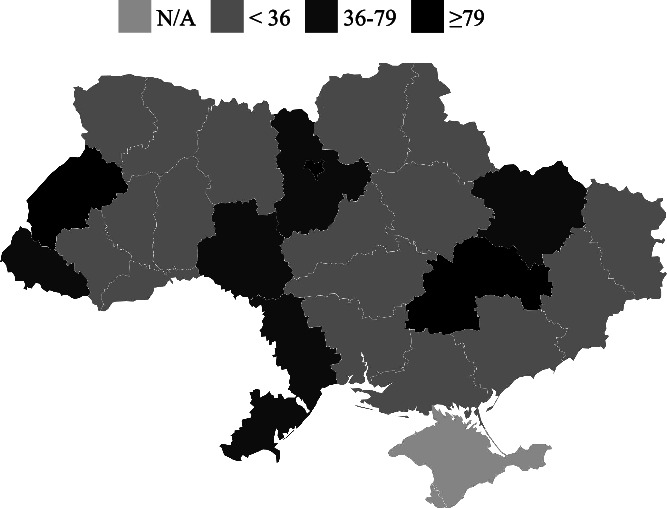
Number of searches in chatbot by oblasts in Ukraine

**Figure 6:**
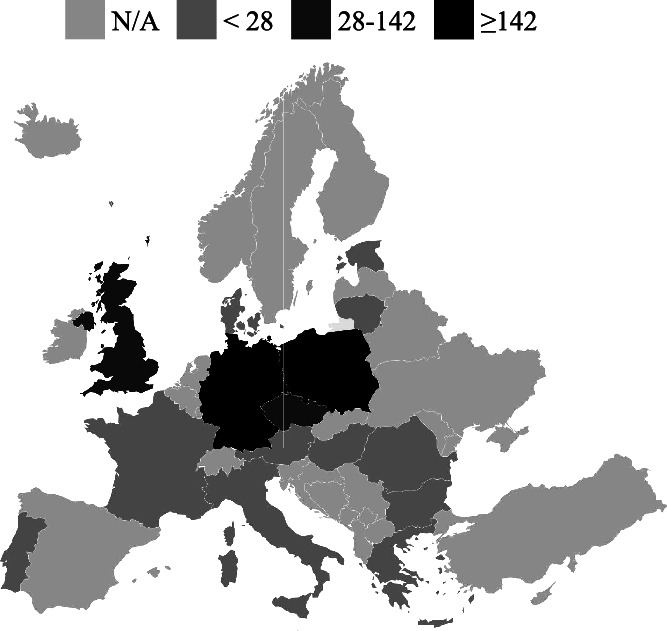
The number of searches in chatbot by users abroad (European countries)

Although 31% of users utilized the search function within Ukraine during the given period, the number of users utilizing the search function outside of Ukraine increased from 11% to 17% by the end of the period. The most commonly used option for obtaining information on the nearest HFs within an oblast in Ukraine was to conduct a search. Conversely, the option to search for the nearest HF in Ukraine using the user’s location was less popular. The highest number of searches was recorded in Kyiv city, Dnipropetrovsk and Lviv oblasts in Ukraine (Fig. 5). Analyzing the distribution of chatbot searches among users in other countries revealed that most users searched in Poland and Germany, with relatively fewer searches in the Czech Republic and the UK (Fig. 6).

At the end of the period, 1433 users utilized the chatbot search function in Ukraine or abroad. Of those, 294 users reported receiving the necessary services through the chatbot.

#### Cross-cutting

Based on the analysis of calls to the HIV/AIDS hotline, it can be noted that all users who went to the website and used the relevant function in the chatbot received the HIV/AIDS hotline contact details. It was recorded that 231 users contacted the HIV/AIDS hotline via the chatbot’s online chat, and 32 used the direct phone call button on the website.

Overall, the DHI received positive feedback with a rating of 4.5 out of 5 stars. Feedback was crucial in addressing challenges promptly. In May 2022, in response to user feedback concerning difficulties in accessing health services abroad, the pertinent features of the DHI were enhanced and broadened. A comprehensive analysis was conducted on a total of 683 feedback messages collected.

Moreover, the DHI was utilized not only by its intended targeted primary user but also by healthcare professionals seeking essential information for their patients. This broadens the potential user base and underscores the need for suitable functionality tailored to healthcare providers, indicating a potential avenue for further enhancement of this DHI.

## DISCUSSION

### Principal findings

The study’s findings are consistent with evidence showing that DHIs extend the reach of care, especially to vulnerable groups during crises [21, 22]. By connecting displaced Ukrainian PLHIV to functioning HFs, the DHI likely prevented dangerous ART interruptions. Furthermore, combining digital products in the DHI increased efficiency, aligning with the findings of another study [23].

Our findings resonate with broader research on digital health in conflict-affected areas. Asi and Williams [36] highlighted the potential of digital health tools to bridge healthcare gaps in conflict-affected areas and contribute to achieving Sustainable Development Goal 3. Similarly to our intervention, they emphasized the ability of digital technologies to overcome physical barriers and support education and outreach efforts. Additionally, our experience in developing and implementing the DHI in Ukraine’s conflict setting echoes the challenges identified by El-Jardali et al. [37] in their scoping review of digital health in fragile states in the Middle East and North Africa region. They similarly noted implementation considerations such as computer literacy, weak technological infrastructure and privacy concerns, which we also encountered and addressed in our intervention.

It is not possible to determine the effectiveness of the DHI due to war-related restrictions; however, it is evident that it played a pivotal role in the national response to the full-scale invasion. In June 2022, the CPH of the MoH of Ukraine prepared a report entitled ‘National response of HIV, TB, HCV, and OST programs to the full-scale invasion.’ The report highlighted that digital solutions have played a significant role in the national response to HIV, tuberculosis (TB), viral hepatitis (HCV) and opioid substitution therapy (OST) programs amidst the war in Ukraine. As detailed in the report, the DHI ‘#ARTporuch’ was identified as an integral component of the national response strategy [5]. Furthermore, in April 2023, the success of the DHI prompted the launch of the ‘#TESTporuch’ initiative aimed at promoting HIV prevention, which was supported by the CPH of the MoH of Ukraine [38].

### Limitations

The study’s limitations encompass the absence of a control group, which hinders the ability to isolate intervention effects. Additionally, reliance solely on usage metrics serves as a proxy for actual health outcomes. Conducting comparative effectiveness research across various solutions and settings would enrich the understanding of optimal crisis-response designs. Moreover, the reliance on data collected through digital platforms may introduce selection bias, as users engaging with the intervention might not represent the entire target population.

It is important to highlight that the ongoing war context may limit the generalizability of the findings to other settings. Furthermore, due to the war, all registries and data sources are closed or restricted, which prevents accurate assessment of the exact number of people who have left the country, migrated internally or lost their lives. This lack of comprehensive data makes it challenging to evaluate the true effectiveness of the intervention in relation to the total PLHIV population affected by the conflict.

The study also lacks quantitative data on the proportion of PLHIV who experienced disruptions in antiretroviral therapy and were subsequently reconnected to HIV treatment through the DHI. This limitation stems from the inability to access or collect such data in the current war situation, where maintaining patient confidentiality and safety is paramount.

### Lessons learned

It is crucial to highlight the lessons learned after implementing the DHI. Primarily, it is essential to acknowledge that in wartime, any open information can be exploited by the aggressor country, necessitating a judicious approach to disseminating information when developing and utilizing publicly accessible DHI. Furthermore, acting expeditiously and implementing DHI functionality in these challenging circumstances was important. In such emergencies, the imperative to act swiftly and implement functionality incrementally takes priority over pursuing perfection and comprehensiveness.

In times of armed conflict, it is vital to be responsive and agile. In particular, the use of agile development methodologies and low-code platforms to rapidly build digital products has proven to be a successful solution when needed by the target population. Also, this ensured flexibility and adaptability to quickly changing conditions. Meanwhile, continuous monitoring of key performance indicators and analysis of user feedback were critical to making experience-based changes and updates to the DHI.

A further lesson learned was that the adaptability of the team responsible for creating and maintaining the DHI was essential. Regular check-ins and meetings were held to monitor results and exchange information about the well-being and support of each team member who could be affected by the ongoing hostilities. This highlights the significance of fostering a supportive and resilient team environment during crises.

Lastly, collaboration with communities and national representatives, such as the CPH of the MoH of Ukraine, allowed us to attract support and implement a large-scale information campaign, which was pivotal in attracting the target population.

Among the challenges, some users refrained from authorizing their phone numbers in the chatbot, indicating reservations. Enhancing trust is an integral part of the information campaign when implementing DHIs for their acceptance. While the requirement for users to authorize with a national phone number in the chatbot is necessary for security, it has the potential to create a barrier for some users. This also underscores the necessity of meticulously balancing security protocols with accessibility, particularly in the context of wartime operations.

It is worth highlighting challenges related to the digital divide. One in ten users requested technical assistance due to digital literacy issues. Furthermore, despite significant progress, Ukraine still has gaps in Internet access in some areas, which have only been exacerbated by the full-scale invasion. As of June 2022, the lack of Internet access in Ukraine varied from 16% to 51%, depending on the oblast [39]. These challenges emphasize the significance of addressing the digital divide, which is also supported by the findings in the countries of the WHO European Region [40]. This also underscores the importance of equity-focused DHI design that addresses the specific needs of low digital literacy users and restricted internet access.

Although no cyberattacks or crimes related to the DHI were identified during the study period, the team prioritized cybersecurity in routine updates of digital products and procedures due to war-related conditions [41].

### Future research directions

Perspectives for further research include exploring the possibilities of integrating such DHIs, assessing their long-term impact, and creating and implementing integrated digital ecosystems to ensure the resilience of health systems in emergencies. Future studies should focus on rigorously evaluating the effectiveness of these interventions in terms of health outcomes, such as continuity of care and treatment adherence among PLHIV in crisis situations.

Particular attention should be drawn to issues of digital divide, trust, digital literacy and other non-technical aspects related to implementing DHIs.

Furthermore, investigating the scalability and adaptability of such interventions to different cultural contexts and types of emergencies would provide valuable insights into global health preparedness.

## CONCLUSION

This case study of the ‘#ARTporuch’ in Ukraine offers insights into the implementation and lessons learned from DHI in reconnecting PLHIV to healthcare during wartime. While quantitative evidence of effectiveness is limited due to the constraints imposed by the wartime context, the study demonstrates the potential of DHIs as a component of crisis-response strategies.

The findings from this study contribute to the growing body of knowledge that can inform the planning of similar interventions in other conflict-affected or resource-constrained settings. This contributes to the ongoing efforts to achieve universal health coverage and enhance health systems resilience planning.

## Supplementary Material

Supplementary_Material_1_Confirmation_of_participation_and_permission_Project_oqaf001

## Data Availability

The data underlying this article cannot be shared publicly due to the sensitive nature of the topic of HIV, the ongoing military aggression, and shelling of critical and civilian infrastructure by the aggressor, which could potentially compromise the safety of the individuals and health facilities involved in the study. The data will be shared on reasonable request to the corresponding author.
