# Supplementary material for: Digital health intervention reconnects war-affected people living with HIV to healthcare: Ukraine case study
Source: Oxf Open Digit Health. 2025 Jan 8;3:oqaf001. doi: 10.1093/oodh/oqaf001 (PMC11998593; doi:10.1093/oodh/oqaf001)
Supplement: Supplementary_Material_1_Confirmation_of_participation_and_permission_Project_oqaf001 [file Supplementary_Material_1_Confirmation_of_participation_and_permission_Project_oqaf001.pdf]

CONFIRMATION OF PARTICIPATION AND PERMISSION TO USE DATA

Funded through the President's Emergency Plan for AIDS Relief (PEPFAR), the "Community Action for HIV Control" Project aims to increase the number of people living with HIV (PLHIV) who know their status and are linked to HIV care, in order to achieve the Joint United Nations Programme on HIV/AIDS (UNAIDS) 95-95-95 goals for HIV epidemic control in Ukraine. The overall goal of the five-year activity is to accelerate Ukraine's efforts to achieve HIV epidemic control by 2030 by improved prevention, testing and linkage to care among key and priority populations.

The Project has developed and implemented many effective innovations, including two chatbots #ARTporuch and #TESTporuch developed in cooperation with the State Institution Center for Public Health of the Ministry of Health of Ukraine.

It is hereby to confirm that Mr Hlib Aleksandrenko was personally involved in the creation of these initiatives and provided invaluable efforts during the management and accomplishment at every stages of the life cycle of the products developed. His responsibilities included product strategy development, creation processes, input data preparation and analysis, testing, implementation, and maintenance, etc.

He is authorized to use depersonalized data, knowledge, lessons learned, and experience information obtained during the implementation of both initiatives for research and educational purposes only with reference to the Project.

Authorized Representative
Chief of Party
Volodymyr Kurpita

January 16, 2024
